# Real-Time Ozone Detection Based on a Microfabricated Quartz Crystal Tuning Fork Sensor

**DOI:** 10.3390/s90705655

**Published:** 2009-07-15

**Authors:** Rui Wang, Francis Tsow, Xuezhi Zhang, Jhih-Hong Peng, Erica S. Forzani, Yongsheng Chen, John C. Crittenden, Hugo Destaillats, Nongjian Tao

**Affiliations:** 1 Biodesign Institute, Arizona State University, Tempe, AZ, 85287-5801 USA; 2 Department of Electrical Engineering, Arizona State University, Tempe, AZ, 85287-5801 USA; 3 School of Sustainable Engineering and the Built Environment, Arizona State University, Tempe, AZ, 85287-5801 USA; 4 Indoor Environment Department, Lawrence Berkeley National Laboratory, Berkeley, CA 94720, USA

**Keywords:** ozone, environmental, epidemiological, population, real-time, selective, sensitive, sensor, wearable, wireless

## Abstract

A chemical sensor for ozone based on an array of microfabricated tuning forks is described. The tuning forks are highly sensitive and stable, with low power consumption and cost. The selective detection is based on the specific reaction of the polymer with ozone. With a mass detection limit of ∼2 pg/mm^2^ and response time of 1 second, the sensor coated with a polymer sensing material can detect ppb-level ozone in air. The sensor is integrated into a miniaturized wearable device containing a detection circuit, filtration, battery and wireless communication chip, which is ideal for personal and microenvironmental chemical exposure monitoring.

## 1. Introduction

Ozone is a reactive constituent of urban atmospheres, associated with asthma and other health effects [1]. In indoor environments, ozone chemistry leads to the formation of potentially harmful secondary pollutants [2–5]. To date, ozone monitoring has been mainly performed by UV photometry [6] with relatively expensive and heavy equipment that needs frequent calibrations. A lower cost portable ozone sensor based on metal oxide has been developed, but it has a high power consumption and is prone to drifts originating from fluctuating ambient oxygen concentrations [7]. An alternative for portable ozone monitor is to use a mass loading Quartz Crystal Microbalance (QCM) which has been developed and tested by Black *et al.* [8]. This system has demonstrated good performance for indoor applications. However, compared to the tuning fork ozone sensor presented in this paper, the QCM-based ozone monitor system is less portable and lacks versatility to simultaneously monitor humidity changes and other pollution-related analytes. Due to the lack of a suitable alternative technique, ozone exposure is currently estimated through measurements performed at regional monitoring stations [9–11]. This approach cannot meet the needs of measuring local ozone level variations, particularly in indoor environments where ozone concentration can be significantly lower than outdoors. It is also unsuitable for assessing personal exposure, which is critical for epidemiologic studies that aim to determine the link between personal ozone exposure and health. Also, in outdoor urban air impacted by motor vehicle emissions, local ozone levels can be partially reduced by its reaction with nitric oxide (NO).

In this work, we describe an ozone sensor based on microfabricated quartz tuning forks (QTF). The sensor is miniaturized with minimum power consumption, and yet can detect ppb-level of ozone concentrations. This design can be ideal for the needs of monitoring local ozone levels and assessing personal ozone exposure.

## 2. Experimental Setup

### 2.1. Sensor array and materials

The QTFs are mechanical resonators with extraordinary mass sensitivity, high thermal and mechanical stability and self-sensing capability [12–15]. The QTFs used in our experiments have a resonance frequency of 32,768 Hz with dimensions of 4 mm, 0.35 mm and 0.6 mm for each prong (Newark In One Electronics). The effective spring constant of these QTFs is ∼20 kN/m and the thermal noise is ∼1 × 10^−4^ nm (root mean square oscillation amplitude of the prongs) at room temperature [12–16]. We built a QTF sensor array of 10 QTFs. Coating QTF sensing elements with different materials allows us to detect multiple analytes [16]. In the present work, the sensing elements include: (1) an ozone sensitive QTF modified with materials based on double carbon bonds with a rapid kinetics towards ozone, (2) a humidity sensitive QTF modified with a neutral hydrophilic polymer to monitor humidity (polyacrylamide), (3) an exposed passivated bare QTF to check the electrical circuit or for changes in the flow rate of the system, and (4) an insulated QTF calibrated to monitor temperature drifts. The six remaining QTF sensing elements are either used for redundant ozone detection or reserved for simultaneous detection of other pollutants (currently under development). In addition to the sensing array, our proposed system includes components to minimize humidity burden (Nafion tubing, 2B Technologies, Inc.), and an ozone scrubber to create ozone-free air for calibration of the device and for reference subtraction to further improve the performance. The air flow is controlled by a 2-way switching valve connected to a minipump (Parker) to deliver air sample to the QTF sensing array (see Section 2.2: Air sample calibration and testing).

Several different ozone sensitive materials, including polybutadiene [8]. (Aldrich), home-made silica nanoparticles [17] modified with butadiene silane and ionic liquid SP-IL100 (gift from Supelco) were tested. Only polybutadiene offered good performance. The amount of the polymer coating was also optimized for best ozone sensitivity. As the ozone reacted with the polymer, a corresponding mass change occurred, resulting in a change of resonant frequency of the QTF.

**Figure 1.**
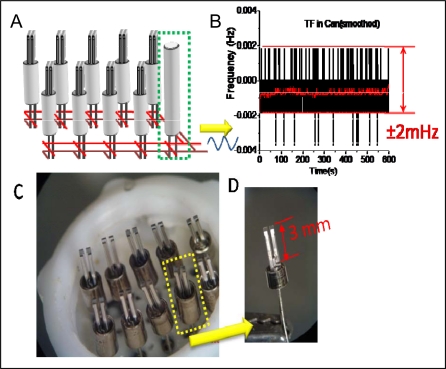
A) Schematic of a QTF sensor array; B) Record of the noise level of the circuit built for the QTF array; C) QTF array with Teflon housing; D) Individual QTF.

The sensor array was housed in a Teflon cell, as shown in Figure 1 [14,15] and driven into oscillation with a circuit, which also monitors the oscillation frequency of each QTF with a mass detection limit of ∼2 pg/mm^2^. The noise level of the circuit was optimized at ∼4 mHz peak-to-peak with a response time of 1 s (1 data/s = 1 Hz) (Figure 1B, black curve) and further decreased to ∼1 mHz peak-to-peak, by smoothing with an equivalent resolution of 0.2 Hz (1 data/5 s) (Figure 1B, red curve). The detection circuit contains further a Bluetooth® chip that can transmit sensor output signals up to 100 meters away to a Bluetooth®-enabled device, such as a laptop or cell phone.

### 2.2. Air sample calibration and testing

Both dry and ambient air samples were used to calibrate the ozone QTF sensors. A UV ozone generator (UVP Corp.) was used to generate samples of different ozone concentrations and a commercial ozone monitor (2B Technologies, Inc) was used for assessment of ozone concentrations of the tested samples. An ozone scrubber provided by 2B Technologies, Inc. was used to build the QTF-sensor array based device with two switching channels, sampling and purging, respectively. The scrubber was included as part of the purging channel.

## 3. Results and Discussion

To demonstrate the selectivity, sensitivity, and robustness of our device for ozone detection, we have completed various experiments for characterization, calibration and ambient testing.

### 3.1. Ozone detection and Tuning Fork sensor calibration

**Figure 2.**
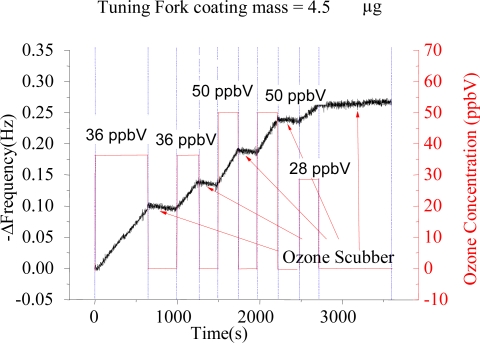
Response of a 4.5 μg polybutadiene coated tuning fork towards alternate low ozone concentration and ozone-free air exposures (alternate time segments are separated in the plot using blue lines). Low ozone concentrations were generated with the UV source, and ozone-free air samples were generated with air samples passing through an ozone scrubber. The exposure was managed through a switching valve, and the actual concentration of ozone was monitored on-line at the outlet of the QTF cell. The slope of the frequency response increased when the sensor was exposed to ozone, and a positive slope (−Δf/Δt) change is indicative of increasing ozone concentrations levels. The response was wirelessly assessed from the device by using a Bluetooth®-enabled laptop.

Figure 2 shows the response of a polybutadiene coated QTF element to different ozone concentrations. The time course of the resonant frequency changes on the QTF sensor (black curve) is compared with ozone concentrations measured with the commercial photometric ozone monitor (red curve). A steady and linear frequency decrease (associated to a mass increase) is recorded when the sensor is exposed to ozone.

**Figure 3.**
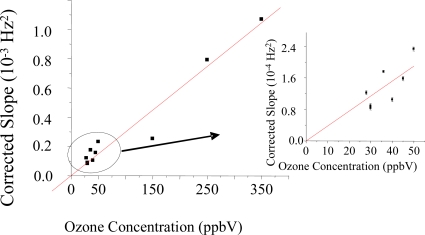
Calibration plots of the response of different tuning fork sensors vs ozone concentration. The inserted figure is the tuning fork response obtained at low ozone concentration range. The response of the sensors (corrected slope) is the frequency slope obtained at a given ozone concentration corrected by subtraction of the frequency slope obtained in presence of ozone-free exposure. The corrected frequency slope is proportional to ozone concentration. A least square linear fitting of the response gives a correlation factor of 3.0 × 10^−6^ ± 1.7 × 10^−7^ Hz^2^/ppbV with 5.6% error, indicating the sensitivity of the response is well-maintained across different ozone QTF sensors, and concentration ranges.

The slope of the linear increase (−Δf/Δt) is proportional to O_3_ concentration (Figure 3). We have studied the origin of the mass increase (see below) and concluded that it is due to the irreversible uptake of oxygen atoms in the reaction of ozone with the sensing materials (see Section 3.3: Characterization of ozone detection reaction products). We have also observed that optimal correlation of the sensor response with ozone concentration is found when the −Δf/Δt measured in the presence of ozone is corrected by subtracting −Δf/Δt measured in the absence of ozone immediately before and after the sample. The last parameter is assessed when the 2-way valve switches and connects the ozone scrubber to the inlet of the sensor array chamber. As a consequence, we evaluated the corrected slope defined as [Δf/Δt (with O_3_) − Δf/Δt (without O_3_)] as the response signal of our ozone sensor. From calibration plots of corrected slope vs. ozone concentration, a correlation factor of 3.0 × 10^−6^ ± 1.7 × 10^−7^ Hz^2^/ppbV (5.6%) is obtained for polybutadiene coating masses ranging between 3.3–6.6 μg, which resulted an optimal coating mass range. We have found this coating mass range to be essential to reach the linear regime of the sensor response (linear −Δf vs Δt, at a given concentration). Lower coating masses produced exponential dependence of −Δf vs Δt as expected by a reaction limited by a pseudo-first order kinetics (not shown), while higher coating masses precluded the QTFs to be driven steadily with our oscillator circuit.

### 3.2. Ozone detection in ambient air

**Figure 4.**
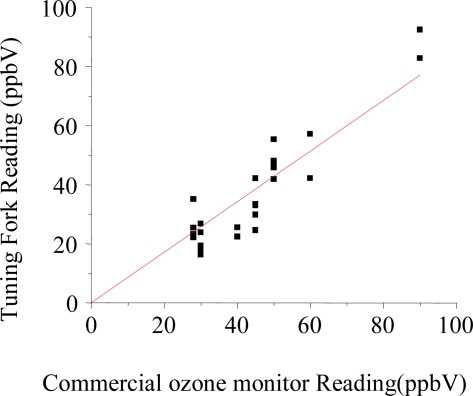
Comparison of ozone level readings obtained from QTF sensors using a calibration plot and function as shown in Figure 3, and readings from a commercial ozone monitor of indoor air samples and artificially ozone spiked samples. The agreement between both methods is 86 %, indicating the ozone QTF sensors have relatively good accuracy. The regression line is again fitted with least square method.

The calibration factors provided good determination of unknown ozone concentrations. Figure 4 shows the correlation between ozone concentrations measured in ambient air by several QTF sensors and the commercial UV ozone monitor (2B Tech Model 202).

The correlation is accurate within 86%. We define the accuracy as the slope of the linear regression line of the tuning fork sensor response vs readings from the commercial ozone instrument. Additionally, the linear regime of the sensor response given by the optimized polymer coating mass on the resonator allowed to use the sensor with repetitive exposure events and alternating low and high ozone levels with a total lifetime of ∼120 ppb-hour. Currently, the ozone sensor has a relatively short lifetime. We are working on improving this aspect. As to the cost of the sensors, a tuning fork cost less than 10 US cents each, while the polymer used is also relatively inexpensive and is commercially available. The modification process is also simple. So we anticipate the cost of the sensor to be very economical.

**Figure 5.**
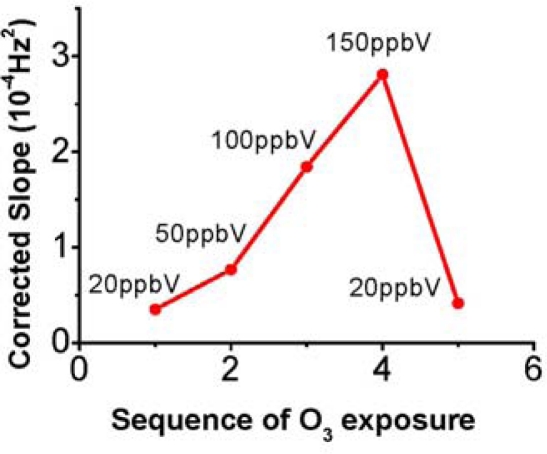
Ozone measurements performed with a single QTF ozone sensor with ozone exposure events equivalent to increasing and decreasing ozone levels observed along a day.

Figure 5 shows the response of the QTF ozone sensor to the variation of ozone level equivalent to a high exposure level during a day.

### 3.3. Characterization of ozone detection reaction products

**Figure 6.**
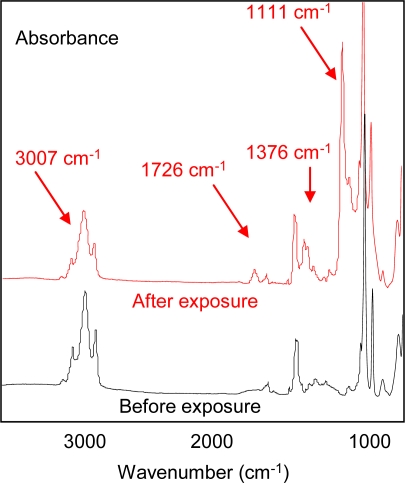
FTIR spectra of a polybutadiene film before and after exposure to 100 ppbV ozone during 2 hours.

In order to understand the sensing mechanism, Fourier Transform Infrared (FTIR) spectroscopic studies were carried out to characterize the reaction products of the alkene-rich sensing material with ozone as well as the reaction kinetics. Ozone reacts with the carbon double bonds (C=C) of polybutadiene, leading to the formation of carbonyl groups and carboxylates. Figure 6 shows the Fourier Transform Infrared (FTIR) spectra of polybutadiene thin film (∼500 nm thick) pre- and post-exposure to 100 ppbV ozone for two hours. The band at 3,007 cm^−1^, corresponding to olefinic C-H stretching, decreased at a much faster rate than the other C-H stretching signatures (at 2,960 and 2,922 cm^−1^), consistent with a preferential attack of ozone on the alkene moiety. Reductions in intensity of alkane C-H stretching signatures at 2,960 and 2,922 cm^−1^ indicated that other oxidation pathways were also present, and/or evaporative losses of small fragments of the polymer produced in the reaction. In addition, new bands were observed at 1,726 cm^−1^ (C=O stretching), 1,377 cm^−1^ and 1,111 cm^−1^ (C–O single bond), indicating buildup of oxygenated functional groups. This uptake of oxygen during ozonation leads to a proportional mass increase of the polymer, which determines the amount of resonant frequency shift of the QTF sensors.

## 4. Conclusions

We have developed a miniaturized ozone sensor using an array of QTFs. QTF sensing elements are integrated with digital control and detection circuit, sample collection and filtration, and wireless communication. The sensor is based on the specific reaction of carbon double bonds of polymer materials, unique sensing capability of microfabricated QTF. We have studied the reaction mechanism of the polymer sensing materials with ozone and determined the sensing mechanism. We have demonstrated real-time and accurate monitoring of ozone at the ppb-level. The QTF sensor is also highly stable, with low cost and low power consumption, which is ideal for personal exposure assessment and protection. Finally, the work demonstrated the importance of integrating novel sensing science with device engineering for real world problems.
